# Case scenario: Management of sexually transmitted infections in primary care based on the fifth edition of the Malaysian guidelines

**DOI:** 10.51866/cpg.916

**Published:** 2025-08-23

**Authors:** Zainal Fitri Zakaria, Rupinder Kaur

**Affiliations:** 1 MBBCh, M.Med Family Medicine, Klinik Kesihatan Seremban, Jalan Rasah, Seremban,Negeri Sembilan, Malaysia. Email: drzainalfitri@moh.gov.my; 2 MBBS, M.Med Family Medicine, Klinik Kesihatan Seksyen 7 Shah Alam, Persiaran Kayangan, Shah Alam, Selangor, Malaysia.

**Keywords:** Sexually transmitted infections, Primary care, Disease management, Malaysian guideline

## Abstract

Sexually transmitted infections (STIs) have a profound effect on sexual and reproductive health, with an estimated 374 million new infections reported globally in 2020. Most STI cases are likely to be presented at primary care clinics, making it crucial for primary care doctors to possess a strong understanding of STIs. Effective management of these infections relies on timely and accurate diagnosis, evidence-based treatment and comprehensive prevention strategies. The Malaysian Guidelines for the Management of Sexually Transmitted Infections Fifth Edition 2024 highlight the importance of treatment, prevention strategies and diagnostic recommendations, underscoring the role of primary care in addressing STIs effectively.

## Introduction

The management of sexually transmitted infections (STIs) in primary care settings faces multiple interconnected challenges that extend beyond mere medical treatment.^1^ Social stigma remains a significant barrier, with many patients reluctant to discuss their sexual health openly, fearing judgement or negative consequences.^1^ This situation is further complicated by healthcare professionals who may lack comprehensive training in STI counselling and management, particularly regarding newer screening methods and resistance patterns.^2^ The rising concern of antimicrobial resistance, especially in diseases such as gonorrhoea, adds another layer of complexity to treatment protocols.^3^ Additionally, primary care facilities often struggle with resource limitations and unclear referral pathways, impacting their ability to provide optimal care.^4^ Successful STI management requires a holistic approach that addresses not only the biological aspects but also the psychological and social dimensions of patient care.^5^

## What is new in the fifth edition?^6^

The fifth edition of the Malaysian Guidelines for the Management of Sexually Transmitted Infections includes several key updates and emphases. These updates aim to provide a comprehensive and current approach to STI management:

- Comprehensive approach: Emphasises the importance of a holistic approach to STI management including prevention, diagnosis, treatment and follow-up care
- Updated treatment recommendations: Highlight any changes or updates in best treatment practices based on current evidence and epidemiological trends
- Improved testing: Emphasises the needs for latest diagnostic and screening tests, including Point-of-Care Testing( POCTs).
- Focus on prevention: Stresses the significance of prevention strategies such as safe sex practices, vaccination (where applicable) and education
- Health promotion: Advocates for health promotion to raise awareness about STIs, facilitate partner notification, reduce stigma and encourage regular testing

## Case scenario

The following case scenario highlights the complexities involved in managing STIs in a primary care setting. Drawing from the fifth edition of the Malaysian guidelines, it provides a comprehensive overview of the diagnostic and therapeutic approaches necessary for effective STI management. References to the guidelines will be cited as needed:

Keith, a 24-year-old man, comes to the health clinic alone and complains of pain on urination with discharge for the past 1 week.

**Question 1: What further history would you like to ask?**

*Answer 1*

- Sexual history – The 6 Ps *(refer to Table 1)*
- Medical history
- Medication history
- Travel history
- Occupation
- Last HIV and STI screening
- Social history (including alcohol intake, smoking and substance abuse) *(refer to page 22, section 3)*

**Table 1:** Components of a sexual history (The 6 Ps).

**i. Partners:** - Gender of sexual partner/s - Number of partners in the last 3 months — not necessary to ask for the specific details (if >5 partners)
**ii. Practices:** - Last sexual intercourse (LSI): - How long ago? - Spouse, regular non-spouse, casual? - If regular, duration of relationship? - Type of sex and use of condoms (oral, vaginal or anal) - In MSM, are they insertive or receptive for anal sex? - How did you meet your partner/s? - Any drug use (self/partner)? - Have you or your sex partner/s ever exchanged sex for life needs (money, housing, safety or drugs)?
**iii. Protection:** - Use of condoms/barrier methods - If all sexual contact was protected, when was the last unprotected vaginal/anal sex? - If not using protection, what are the reasons?
**iv. Past history:** - Known STI/symptoms (diagnosis, when and whether it was treated) - Known STI/symptoms in the partner/s (diagnosis, when and whether it was treated) - Previous sexual intercourse with a different partner (for the last 3 months) — to obtain the same information as the LSI
**v. Pregnancy planning:** - Any plans/desires to have children/more children? - Assess timing, emphasise the importance of prevention and conduct preconception education.
**vi. Plus (pleasure, problems and pride):** - How is your sex life? - What difficulties are you having with your sex life or during sex? - What support do you have about your gender identity and/or sexual orientation?

**Question 2: What specific questions would you ask regarding his symptoms?**

*Answer 2*

Ask for specific genital problems *(refer to page 25: Symptoms review in men)*

- Urethral discharge
- Dysuria
- Genital skin problems
- Testicular pain/swelling and ejaculatory problems
- Peri-anal/anal symptoms (in MSM)

You gathered that Keith is generally healthy, with no significant past medical conditions, no history of STIs and no known drug allergies. He does not use recreational drugs and identifies as heterosexual. He reports a recent history of unprotected sexual intercourse with someone he met on social media. He also has a history of multiple sexual partners, mostly sex workers and work colleagues. Keith works as an IT officer in a reputable firm.

One week ago, Keith noticed a white, purulent discharge from his penis after waking up in the morning. The discharge became progressively more noticeable, and he also started experiencing pain during urination (dysuria). He also noted mild discomfort in his lower abdomen and some swelling in his groin area.

*Recent symptoms (past 3 days)*

Over the past 3 days, Keith had developed systemic symptoms, including a low-grade fever of approximately 38°C and increased fatigue. He had also began experiencing joint pain and swelling in both knees, affecting his ability to walk comfortably. In the last 24 hours, he noticed red, tender lesions on his hands and feet, particularly around the fingers and toes.

**Question 3: What physical examination would you do?**

*Answer 3*

- General examination with vital sign assessment
- Genitourinary examination
- Joint examination
- Skin examination
- Neurological examination

On examination, Keith has a temperature of 38.5°C, appears fatigued and is in mild distress. He presents with purulent urethral discharge and tenderness over the pubic region, without significant swelling or lymphadenopathy. There is bilateral knee swelling with erythema and pain on range of motion. Multiple small, erythematous pustules are noted on the palms of the hands and soles of the feet, along with a few painful ulcers. There are no signs of meningitis or central nervous system involvement.

**Question 4: What would be your differential diagnoses at this point?**

*Answer 4*

1. Gonococcal urethritis (likely *Neisseria gonorrhoeae infection)*
2. Disseminated gonococcal infection (DGI)
3. Chlamydia infection (although less likely due to purulent discharge)
4. Reactive arthritis (although DGI is a more likely cause of arthritis in this case)
5. Septic arthritis due to other causes (e.g. staphylococcal infection)

**Question 5: What investigations would you do at your centre?**

*Answer 5*

STI screening for heterosexual men

- Blood test: HIV Ag/Ab, syphilis serology (RPR/VDRL), HBsAg and anti-HCV
- Urethral swab for Gram staining and C/S
- Urine: Chlamydia/gonorrhoea (CT/NG) NAAT if available *(refer to page 25, subsection 2.2)*

Since Keith is experiencing joint swelling and skin lesions, you suspect he has DGI and decide to call an ID physician for joint aspiration and culture of skin lesions and hospitalisation. The ID physician obliged to see him at the clinic on the same day and will decide if he needs admission.

While writing your referral letter, the laboratory technologist reports the presence of intracellular gram-negative diplococci on microscopy; RPR and HIV RDT are negative.

**Question 6: How would you treat him?**

*Answer 6*

Keith will require IM/IV ceftriaxone for at least 7 days if confirmed to have DGI. However, in this case, treatment will be initiated at the hospital after collecting additional specimens, including a pharyngeal swab, blood cultures with sensitivity testing, synovial fluid aspirate for Gram staining, culture and NAAT and skin lesion samples for culture and NAAT *(refer to page 72: Section Management, subsection* i).

**Table 2:** Pharmacological treatment.

Type of Infection	Preferred	Alternative
Uncomplicated (urogenital and anorectal)	Ceftriaxone 500mg IM STAT (Use 1g if weight more than 150kg)	Gentamicin 240mg IM STAT AND Azithromycin 2g PO STAT
Uncomplicated (pharynx)		Discuss with expert
Gonococcal conjunctivitis	Ceftriaxone 1g IM STAT *one-time lavage of the infected eye with saline should be considered	Refer Ophthalmologist
Disseminated Gonococcal Infection (DGI) *(Hospitalisation and physician consultation are recommended)*	Ceftriaxone 1g IM/IV daily Duration of parenteral treatment: At least 7 days for DGI with arthritis- dermatitis syndrome 10-14 days for DGI with meningitis 4 weeks for DGI with endocarditis	Refer relevant subspecialties

**Question 7: What advice would you give Keith?**

*Answer 7*

- He should inform his sexual partner(s) so they can undergo appropriate testing and receive treatment if necessary.
- He should abstain from sexual intercourse until completion of therapy and follow-up testing.
- Sex partners should be instructed to abstain from condomless sexual intercourse for 7 days after they and their sex partners have been treated and after resolution of symptoms.
- Educate on and promote condom use and provide condoms *(refer to page 72: Section Management, subsection ii*).

One week post-treatment, Keith returns to the clinic. His fever has resolved, and his symptoms of arthritis and skin lesions have significantly improved. He is now able to walk comfortably with minimal joint pain. The urethral discharge has resolved. Based on his hospital discharge summary, he was diagnosed with DGI; his synovial fluid aspirate was positive for gonorrhoea, but skin vesicle culture is pending. Pharyngeal swab for gonorrhoea was negative. His sexual partners have been tested and treated. The case was notified under the Infectious Disease Act 342. The Infectious Disease Act 342, officially known as the Prevention and Control of Infectious Diseases Act 1988 (Act 342), is a Malaysian law enacted to provide for the prevention and control of infectious diseases in Malaysia. It empowers the Ministry of Health to take necessary measures to prevent and control the spread of infectious diseases. Certain diseases (e.g. tuberculosis, dengue, HIV, gonorrhoea and COVID-19) are classified as notifiable, meaning healthcare providers must report cases to health authorities. Under this Act, individuals can be legally required to undergo testing, isolation or treatment if diagnosed with or suspected of having an infectious disease. In cases of STIs, such as gonorrhoea, the law may mandate partner notification and treatment to control disease spread. Non-compliance with the provisions (e.g. refusing testing or quarantine or giving false information) can result in fines or imprisonment.^7^

**Question 8: Does Keith require a test of cure (TOC)?**

*Answer 8*

No, he does not require a TOC. A TOC for gonorrhoea is a repeat test performed 1-2 weeks after treatment to check for persistent infection. It is recommended in certain situations, including the following:

- Non-standard treatment if a non-standard treatment regimen is used
- Antibiotic resistance if antibiotic susceptibility testing indicates resistance to ceftriaxone or azithromycin
- TOC for pharyngeal gonorrhoea because of the risk of treatment failure *(refer to page 73: Section Management, subsection iii*)

Three weeks post-treatment, Keith has made a full recovery. No further joint pain or skin lesions are noted. He has not been involved in any sexual activity since the past 3 weeks but is keen to resume his usual routine and sexual practices.

**Question 9: Does Keith need follow-up?**

*Answer 9*

Yes, he should be followed up with a repeat HIV test in case he is in the window period, and STI screening should be performed routinely if his risky behaviour persists. He should also be offered PrEP once HIV has been ruled out.

**Question 10: Any other advice you would give to Keith?**

*Answer 10*

He should be advised on steps to reduce STI exposure:

- Abstinence is the only way to completely avoid STIs.
- Get vaccinated for hepatitis B and HPV.
- Reduce the number of sexual partners.
- Undergo regular testing: He and his partners should get tested and share the test results.
- Be in a mutually monogamous relationship with a partner who has been tested and does not have an STI.
- Use condoms appropriately every time he has sex.

## Conclusion

This case demonstrates the multifaceted challenges of managing STIs in primary care, where the patient’s recovery from DGI relied on prompt identification, accurate diagnosis and comprehensive treatment. It highlights the necessity of addressing both clinical and social dimensions - including partner notification, health education and guideline adherence - while emphasising the value of structured follow-up care and preventive counselling. By implementing this holistic approach, primary care providers can optimise individual outcomes and contribute to reduced community transmission rates.
